# Harnessing machine learning to predict prostate cancer survival: a review

**DOI:** 10.3389/fonc.2024.1502629

**Published:** 2025-01-10

**Authors:** Sungun Bang, Young Jin Ahn, Kyo Chul Koo

**Affiliations:** Department of Urology, Gangnam Severance Hospital, Yonsei University College of Medicine, Seoul, Republic of Korea

**Keywords:** artificial intelligence, machine learning, prostate cancer, survival, precision medicine

## Abstract

The prediction of survival outcomes is a key factor in making decisions for prostate cancer (PCa) treatment. Advances in computer-based technologies have increased the role of machine learning (ML) methods in predicting cancer prognosis. Due to the various effective treatments available for each non-linear landscape of PCa, the integration of ML can help offer tailored treatment strategies and precision medicine approaches, thus improving survival in patients with PCa. There has been an upsurge of studies utilizing ML to predict the survival of these patients using complex datasets, including patient and tumor features, radiographic data, and population-based databases. This review aims to explore the evolving role of ML in predicting survival outcomes associated with PCa. Specifically, we will focus on the applications of ML in forecasting biochemical recurrence-free, progression to castration-resistance-free, metastasis-free, and overall survivals. Additionally, we will suggest areas in need of further research in the future to enhance the utility of ML for a more clinically-utilizable PCa prognosis prediction and treatment optimization.

## 1 Introduction

Prostate cancer (PCa) is a diverse disease for both patients and treatment providers. Most PCa is indolent in nature; however, a subset of patients suffer from aggressive and metastatic disease. In a single prostate, PCa is heterogeneous among lesions, and it also varies between individuals in terms of genetic mutations (1). The treatment strategy is primarily influenced by prognosis and survival predictions. Decisions are guided by socioeconomic factors, such as age, overall health, and life expectancy, as well as clinical factors, including the Gleason grade, TNM staging, and prostate-specific antigen (PSA) levels (2, 3).

In the age of precision medicine, personalizing patient care is crucial for choosing the most effective treatment option. Current PCa treatment guidelines rely on risk stratifications derived from conventional linear models, including survival analysis and the Cox-proportional hazard model. However, due to the complex, nonlinear interactions among various prognostic factors in PCa biology, using only these linear methods may make predicting individual survival outcomes challenging. Artificial intelligence (AI) and machine learning (ML) methods are capable of processing large amounts of data in a comparatively short amount of time and, therefore, are increasingly being used in the medical field. ML could be used in drug discovery, gene expression profile studies, biomarker studies in multi-omics panel construction and analysis, and digital pathology slide analysis (4, 5).

**Figure 1:**
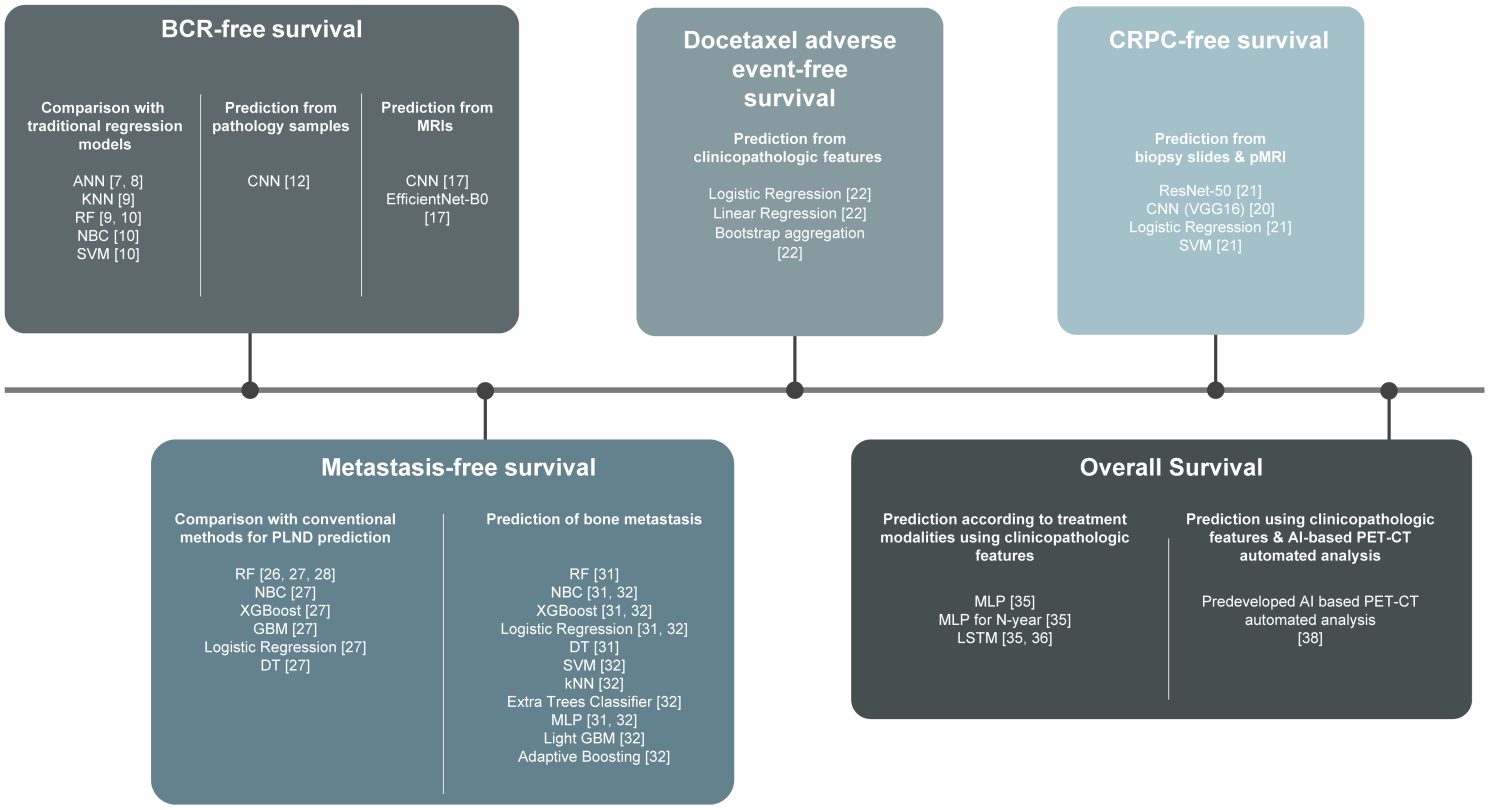
Harnessing machine learning to predict prostate cancer survival: a comprehensive review. ANN, Artificial Neural Networks; kNN, k-Nearest Neighbor; RF, Random Forest Classifier; NBC, Naïve Bayes Classifier; SVM, Support Vector Machine; CNN, Convoluted Neural Network; XGBooost, Extreme Gradient Boosting; GBM, Gradient Boosting Machine; DT, Decision Tree; MLP, Multilayer Perceptron; LSTM, Long Short-Term Memory.

In the era of personalized management, there have been efforts to predict PCa survival using ML algorithms developed based on individual data. In this review, we present contemporary studies that have investigated prognostic algorithms regarding biochemical (BCR)-free survival, castration resistance-free survival, complication-free survival, metastases-free survival, and overall survival (OS) and how these study outputs may be translated into optimal clinical practice. We also provide future outlooks based on the clinical implications of these studies (**Figure 1**).

## 2 BCR-free survival

### 2.1 Comparison with traditional regression models

BCR is defined as an elevation of PSA following primary definitive therapy such as radical prostatectomy or radiation therapy, indicating recurrence of PCa and, therefore, requires salvage radiotherapy (6). Efforts to predict BCR-free survival using AI has started as early as in 2001.

Tewari et al. described artificial neural networks (ANN) to predict disease recurrence or BCR in clinically localized PCa after radical prostatectomy. It shows probability estimates for recurrence at a certain given follow-up time by using clinical available variables instead of depicting survival curves. They analyzed 1,400 patients who underwent standard pelvic lymphadenectomy and radical prostatectomy, with follow-up for at least one year. By employing ANN, they achieved more accurate predictions compared to multivariate statistical models. The ANN demonstrated a sensitivity of 55%, specificity of 90%, positive predictive value of 76%, negative predictive value of 82%, and an overall accuracy of 76%. For regression analysis, these values were 15%, 94%, 64%, 64%, and 66%, respectively. It is noteworthy that the ANN model did not provide survival curves but only estimated BCR recurrence rates at a specific follow-up time after treatment, based on preoperative variables such as age, race, serum PSA, biopsy Gleason score, and systemic biopsy-based staging (7, 8).

Wong et al. analyzed 338 patients who had undergone radical prostatectomy by a single surgeon. They adopted K-nearest neighbor, logistic regression, and random forest classifier as ML models and examined the predictability of BCR at one-year post radical prostatectomy and compared it with that of traditional Cox regression analysis. Despite the fact that the models were generated from a relatively small population with limited follow-up and its database, all three AI models (K-nearest neighbor (area under the curve [AUC] = 0.903), random forest tree (AUC = 0.924) and logistic regression (AUC = 0.940) outperformed the traditional classic Cox regression analysis (AUC = 0.865), suggesting that AI models predicting survival rates can outperform traditional models, and thus, appropriate for a futuristic approach towards precision medicine (9).

Tan et al. analyzed 1130 patients who underwent radical prostatectomy with a median follow-up of 70.0 months. 176 (15.6%) patients developed BCR at a median time of 16.0 months (interquartile range [IQR] = 11.0 – 26.0). Notably, for predicting a 5-year BCR, Naïve Bayes and random forest tree models achieved AUC values of 0.894 and 0.888, respectively, outperforming all three conventional models, which achieved AUC values of 0.799, 0.749, and 0.750 for KATTAN, CAPSURE, and JHH, respectively. The support vector machine model, with an AUC of 0.855, showed comparable performance to the three conventional models. Further external validation with a separate demographic cohort would be needed to confirm the generalizability of the findings (10).

### 2.2 BCR-free survival prediction from pathology samples

The interpretation of pathological samples is a good candidate for AI technology application since AI algorithms can be utilized to identify and quantify cancer cells, Gleason score, tumor length, tumor proportion, grade group, perineural invasion, cribriform pattern, and intraductal features (4).

From a nested case-control study of 685 patients from Johns Hopkins, Pinckaers et al. developed a novel deep-learning-based biomarker developed by Convoluted Neural Network (CNN) and ResNet50-D with tissue microarray hotspots of post-prostatectomy pathology samples and validated in an independent cohort of 204 patients from New York Langone Medical Centre. An odds ratio of 3.32 (confidence interval [CI] 1.63 – 6.77; *p* = 0.001) per unit increase was obtained from the nested case-control study, matched on Gleason sum, age at surgery, race, and pathologic stage. Additionally, a hazard ratio (HR) of 3.02 (CI 1.10–8.29; *p* = 0.030) per unit increase was obtained from the external validation cohort, adjusted for International Society of Urological Pathology grade, pathological stage, preoperative PSA level, and surgical margins status. Thus, their deep learning-based marker provided a continuous score according to the velocity of BCR. However, the marker was based on tissue microarray, which is a limited sample of the entire tumor lesion, and thus, more aggressive cancer patterns could potentially be present outside of the sample. Future validation would be needed on the entire prostatectomy sections and across various cancer foci (11).

Sandeman et al. devised an AI algorithm that can predict post-prostatectomy outcomes from biopsy samples. 516 slides from 331 patients were used in the training set, and 2,088 slides from 391 patients were used in the independent control set. Clinical information from electronic surgical pathology reports such as extracapsular extension, seminal vesicle invasion, nodal status, and pathologic stage were collected, and hematoxylin and eosin (H&E) biopsy slides were scanned with a Panoramic 250 Flash III scanner. This information was trained by two independent convolutional neural networks (CNNs) for multi-class semantic segmentation of tissue (CNN-T) and Gleason grades. In the validation cohort, the model detected cancer with a sensitivity and specificity of both 98%. Among them, Grade group 3–5 PCa had an increased risk for BCR compared to Grade group 1–2 (HR = 5.91; 95% CI 1.96 – 17.83). Indeed, external validation is warranted, taking into account that biopsy techniques and tissue preparation methods may vary by institution (12).

### 2.3 BCR-free survival prediction from magnetic resonance images

Prostate magnetic resonance imaging (MRI) provides data regarding PCa, such as tumor size, extracapsular extension, seminal vesicle invasion, and pelvic lymph node metastasis (PLNM). Radiomics from prostate MRI can provide predictive information regarding post-prostatectomy outcomes.

Hou et al. devised a deep survival network based on MRI radiomics using clinical MRI and histopathologic data from 579 pathologically diagnosed PCa patients at a single tertiary center, with 463 patients in the training set and 116 patients in the test set. The primary endpoint was BCR-free survival probability utilizing their novel MRI radiomics signature (RadS). Then, two AI-derived predictions were measured to detect T3 and PLNM using two predefined studies with clinicopathological variables (13, 14). Finally, to predict BCR-free survival, a multimodal integrative deep survival network known as iBCR-Net was developed by combining RadS, AI-predicted T3 stage, and AI-predicted PLNM with 17 indicators from clinical, radiological, and pathological data. In comparison to traditional methods, such as the D’Amico score, Cancer of the Prostate Risk Assessment (CAPRA) score, and CAPRA post-surgical score, the iBCR-Net demonstrated up to 5.16-fold, 12.8-fold, and 2.09-fold improvements in prediction accuracy, respectively (*p* < 0.05 with the log-rank test). Notably, the study was retrospective, limited to a single institution with a short-term follow-up, and patients with high recurrence rates might have been excluded (15).

Lee et al. analyzed 437 patients who underwent post-radical prostatectomy mpMRI with a median follow-up of 61 months. The prostate MRI radiomics deep learning model, which included 17 layers of convolution, was combined with six clinical parameters and showed superior performance compared to conventional multiparametric MRI-based radiomics approaches. Bourbonne et al. conducted a retrospective analysis of 195 patients at high risk for PCa recurrence, with a median follow-up of 46.3 months. They used a radiomics model based on MRI T2 and apparent diffusion coefficient (ADC) maps to predict BCR-free survival after radical prostatectomy (16). Their model’s performance was lower (HR 6.8) compared to Lee’s model (HR 7.7) (17).

Similarly, Li et al. employed a radiomics approach with 198 patients and a median follow-up of 35 months, but their model showed a lower prognostic performance (C-index = 0.77) compared to Lee’s model (C-index = 0.89) (18). Further studies could potentially incorporate prostate-specific membrane antigen (PSMA) positron emission tomography (PET) radiomics into the deep learning algorithms for more accurate predictions. The aforementioned studies that have investigated BCR-free survival using ML methods are summarized in **Table 1**.

**Table 1:** BCR-free survival using ML methods.

Reference	Year	AI Model	Patients	Parameters	Predicted Outcomes
Tewari et al. (7)	2004	ANN	1400 PCa patients who underwent RP	Clinicopathological features	AUROC = 0.83, sensitivity = 85%, specificity = 74%
Tewari et al. (8)	2001	ANN	1400 PCa patients who underwent RP	Clinicopathological features	ANN predicted BCR better than multivariate statistical models. Overall accuracy, sensitivity, specificity = 76%, 55%, 90% vs 66%, 15%, 94%, respectively.
Wong et al. (9)	2019	kNN, RF, logistic regression	338 PCa patients who underwent RP	Clinicopathological features	ML models outperformed the Cox regression model, showing better accuracy (AUC = 0.903-0.940 vs. 0.865).
Tan et al. (10)	2022	NBC, RF, SVM	1130 PCa patients who underwent RP	Clinicopathological features	For predicting a 5-year BCR, NB and RF outperformed conventional models (AUC = 0.894 and 0.888 vs. 0.749-0.799).
Pinckaers et al. (11)	2022	PyTorch	685 PCa patients who underwent RP/validation on 204 PCa patients	Clinicopathological features, DLS biomarker from TMA spots	Odds ratio of 3.32 (CI 1.63–6.77; *p* = 0.001) per unit increase obtained from the nested case-control study, matched on Gleason sum, age at surgery, race, and pathologic stage.
Sandeman et al. (12)	2022	CNN-T(tissue), CNN-GG(Gleason Grade)	750 PCa patients (331 patients for training set, 391 patients for validation set)	Clinicopathological features	In the validation cohort, the model detected cancer with a sensitivity of 98% and specificity of 98%. Grade group 3–5 PCa had an increased risk for BCR compared to Grade group 1–2 (HR = 5.91; 95% CI 1.96 – 17.83).
Hou et al. (15)	2021	iBCR-Net using Cox-GBM, Cox-DL, N-MTLR	579 PCa patients (463 for training set, 116 for test set)	Clinicopathological features, MRI radiomic features (RadS), AI predicted ECE, AI predicted PLNM	iBCR-Net showed 2.09-12.8 fold benefit (*p* < 0.05 with the log-rank test), compared to conventional methods for BCR-free survival prediction.
Lee et al. (17)	2023	EfficientNet-B0, CNN	437 PCa patients who underwent RP	MRI Radiomic features	Better performance than previously suggested models, such as Bourbonne’s model (16) and Li’s model (18).

ANN, Artificial Neural Networks; kNN, k-Nearest Neighbor; RF, Random Forest Classifier; NBC, Naïve Bayes Classifier; SVM, Support Vector Machine; CNN, Convoluted Neural Network; GBM, Gradient Boosting Machine.

## 3 CRPC-free survival

The primary treatment modality for advanced PCa is androgen deprivation therapy (ADT). Patients who exhibit a favorable response to ADT are known as castration-sensitive PCa (CSPC). However, a subset of these patients progresses to castration-resistance, experiencing a decline in response to ADT and disease progression, leading to a dismal prognosis (19). Therefore, it is crucial for clinicians to detect CRPC in a timely manner to administer second-line therapies such as androgen receptor axis targeting agents (ARAT) or chemotherapies, potentially increasing OS.

Nakata et al. examined 180 metastatic hormone naïve prostate cancer (HNPC) patients who initially received combined androgen blockade, and developed a deep learning algorithm (DLA) using patients’ prostate needle biopsy H&E patch images. First, they performed multivariate analysis and noted that time to CRPC was the most significantly associated factor associated with OS (*p* < 0.001), even more strongly associated than having a Gleason score ≥8. Thereafter, they selected two groups by time to CRPC >24 months (n = 18) and <6 months (n = 6) and applied CNN to construct an AI-based DLA. Sixteen other metastatic HNPC patients were used as an external validation set (hormone-sensitive group: n = 8, non-hormone-sensitive group: n = 8). The ratio of hormone-sensitive patches to all patches was significantly different between the two groups (*p* = 0.015; median 0.575 for hormone-sensitive group vs 0.708 for non-hormone-sensitive group), thereby confirming the DLA with the external validation set. Even though this study was based on a small number of cases from a single institution with only a Japanese population, it is notable that CSPC patients with time to CRPC > 24 months, 5-year OS was 96.7%, thereby not requiring upfront treatment (20).

Zhou et al. formulated a joint model integrating prostate MRI, prostate biopsy H&E slides, and ML using data from three medical centers. The data of 140 eligible patients at center A was used for a training set, and the data of 61 eligible patients from centers B and C was used as an external validation set. Regions of Interest (ROI) was annotated at T2-weighted imaging, diffusion-weighted imaging, and ADC. The ResNet-50 was the most superior of all in predicting CRPC progression, with an AUC of 0.887 and 0.768 in training and test sets, respectively. This retrospective study had incomplete clinical data for many patients and therefore excluded key clinical prognostic factors, which was a limitation of the study. Further large-scale studies utilizing multimodal ML would be warranted to ensure the utility of the developed algorithm (21). The aforementioned studies that have investigated CRPC-free survival using ML methods are summarized in **Table 2**.

**Table 2:** CRPC-free survival using ML methods.

Reference	Year	AI Model	Patients	Parameters	Predicted Outcomes
Nakata et al. (20)	2022	VGG16 (a type of CNN)	180 mCSPC patients who initially received CAB/16 patients for external validation	Clinicopathological features, H&E images	Ratio of hormone-sensitive patches to all patches significantly different between the two groups (*p* = 0.015; median 0.575 for hormone-sensitive group vs 0.708 for non-hormone-sensitive group). For CSPC patients with time to CRPC > 24 months, 5 year OS was 96.7%.
Zhou et al. (21)	2024	LR, SVM, ResNet-50	140 PCa patients (training set)/61 patients (test set)	Clinicopathological features, MRI radiomics by PyRadiomics	ResNet-50 was the most superior of all in predicting CRPC progression, with an AUC of 0.887 and 0.768 in training and test sets, respectively.

CNN, Convoluted Neural Network; LR, Logistic Regression; SVM, Support Vector Machine.

## 4 Complication-free survival

When choosing agents for advanced prostate cancer, fundamental regimens include ADT, ARAT, docetaxel, poly (ADP-ribose) polymerase (PARP) inhibitors, and pembrolizumab. Alternative regimens vary by stage and risk groups for the treatment of PCa (19).

Deng et al. used data from three different cohorts, with a combined total of 1,600 PCa patients in phase III clinical trials, to evaluate the factors that can predict docetaxel discontinuation due to adverse events (22). The investigators evaluated the prediction performance of five base learners: linear regression, logistic regression, Cox regression, bootstrap aggregation with classification and regression trees (23), and random forest (24), using adverse events as the study endpoint. Random forest achieved the highest AUC score in both the full dataset and across the three cohorts, with a median AUC of 0.627, leading the investigators to select random forest as their base learner. The top ten important features identified included albumin, sodium, total protein, magnesium, testosterone, neutrophil count, white blood cell count, phosphorus levels, and medical history related to vascular disorders and social circumstances. Their algorithm, when tested in the validation cohort, demonstrated an AUC of 0.190, significantly higher than the random baseline of 0.104 (*p* = 0.003). This indicated that within the total 1,000 metastatic CRPC patients, 104 patients were wrongly assigned to docetaxel chemotherapy, and that the utilization of the developed algorithm could have saved approximately ten patients. This study highlights the potential for personalized assessment in predicting treatment discontinuation. However, missing values raised concerns regarding the integrity of the data. Additionally, in recent years, Extreme Gradient Boosting (XGBoost) and Light Gradient-Boosting Machine have become more powerful tools for training tree-based models, and their use could potentially outperform the RF model.

## 5 Metastasis-free survival

### 5.1 Lymph node metastasis-free survival

Pelvic lymph node dissection (PLND) or extended PLND (ePLND) is recommended for intermediate-risk patients with an estimated survival of more than ten years, as well as for high- and very high-risk patients. PLND can provide accurate staging and risk stratification and help determine the need for adjuvant treatment. However, performing PLND requires additional operative time and can subsequently lead to complications such as lymphocele formation (25). Therefore, predicting metastasis-free survival for localized PCa is crucial in deciding the extent of treatment, such as whether to treat LNs during radical prostatectomy or radiotherapy.

Nomograms have been suggested to predict preoperative LN invasion (LNI) status, and the Memorial Sloan Kettering Cancer Center (MSKCC) nomogram is one of the most widely used in clinical practice. Hou et al. investigated whether traditional nomograms could be improved with ML assistance. With the data of 248 patients treated with radical prostatectomy with either ePLND or PLND, their novel ML-assisted model was compared with the MSKCC nomogram. The random forest-based model exhibited highest AUC of 0.906 (95% CI 0.856 – 0.928) among the developed ML-assisted models. Additionally, the developed model with a 5-15% cutoff was superior to the MSKCC nomogram, especially for a 10% cutoff which spared 47.2% of ePLNDs while missing only 1.7% of LNIs. The authors concluded that precisely defined MRI characteristics, such as ADC, D-max, PI-RADS v2 score, MRI-reported T and N stages, were significant predictors of LNI, contributing to the improved accuracy over the MSKCC nomogram. Limitations were small sample size and potential bias in patient selection.

Hou et al. also developed a novel pelvic LN metastasis risk model by integrating radiomics of MRI images, deep transfer learning representation, clinical data, biopsy findings, MRI reports by radiologists, and compared with the MSKCC and Briganti nomograms for predicting LN metastasis-free survival. The PLNM-Risk calculator was developed using an open-source AutoGluon platform. A total of 1,843 patients from two tertiary care medical centers, and 401 patients were used after patient selection. Center 1 data of 280 patients were used for the training set, and data of 71 patients were used for the internal testing set. Center 2 data from 50 patients were used for external testing. The pelvic LN metastasis model could have spared 59.6% of ePLNDs at the cost of missing only 1.7% of pelvic LN metastasis cases, outperforming the current MKSCC and Briganti nomograms. The developed model exhibited higher predictability for pelvic LN metastasis risk. However, due to the small number of subjects, further validation with a larger number of patients from a heterogeneous population would be warranted before being replaced with contemporary nomograms (26).

Wang et al. established a LN metastasis prediction model in intermediate- and high-risk PCa patients. They analyzed 24,470 patients from the SEER database using ML algorithms. In a multivariate logistic analysis, T stage, PSA level, Gleason score, and the presence of bone metastasis were identified as independent predictors of LN metastasis in these patients. The study then assessed the prediction performance of six machine learning algorithms–random forest, naive Bayesian classifier, XGBoost, gradient boosting machine, logistic regression, and decision tree–using both training and test sets. The gradient boosting machine model demonstrated the highest prediction performance, with an F1 score of 0.838 and an AUC of 0.804. This led to the development of a preliminary regional LN metastasis risk calculator for intermediate- and high-risk PCa patients (27).

Quantitative PSMA PET analysis can detect metastatic targets that are undetected with conventional imaging such as MRI and computed tomography scans. Cysouw et al. conducted a prospective study involving 76 patients with intermediate- and high-risk PCa. The patients underwent a preoperative [^18^F]DCFPyL PET-CT, followed by robot-assisted radical prostatectomy with ePLND. Radiomic features were extracted from the delineated tumors from PSMA-PET images and the random forest ML was used to generate the algorithm. The model predicted LN invasion (AUC 0.86 ± 0.15, *p* < 0.01), nodal or distant metastasis (AUC 0.86 ± 0.14, *p* < 0.01), Gleason score (0.81 ± 0.16, *p* < 0.01), and ECE (0.76 ± 0.12, *p* < 0.01). AUCs were higher compared to standard PET metrics. These findings indicate the association between PSMA expression on PET and primary tumor histology, as well as metastatic tendency. Limitations include a small dataset size and the absence of external validation (28).

### 5.2 Bone metastasis-free survival

In an autopsy study, approximately 90% of men who died of PCa metastases were diagnosed with metastases to the bone (29). PCa has a tropism for metastasizing to bones, and therefore, most PCa patients with BM experience skeletal-related events (SREs), including pathologic fracture, spinal cord compression, and hypercalcemia. SREs in PCa reduce quality of life and worsen survival. Predicting candidates who are at a high risk of developing BM allows for early intervention in order to potentially delay or prevent SREs, such as treatment with the RANK ligand inhibitor denosumab and the bisphosphonate zoledronic acid (30).

Liu et al. retrospectively analyzed 207,137 PCa patients from the SEER data, of whom 6,725 (3.2%) developed bone metastasis. Gleason score, PSA level, T and N stage, and age were positively associated with bone metastasis. They used six different ML algorithms, including decision tree, random forest, logistic regression, naïve Bayes classifier, XGBoost, and the Multilayer Perceptron, to build prediction models. The XGBoost model had the best predictive power (AUC = 0.962, accuracy = 0.884, sensitivity (recall rate) = 0.906, and specificity = 0.879). An XGBoost model-based web predictor was developed for bone metastasis risk in PCa patients and was posted at: https://share.streamlit.io/liuwencaincu/prostate-cancer/main/prostate.py. Limitations included the inclusion of only initial diagnostic information, without sequential therapeutic information, which could affect survival. A larger volume of external validation is also needed (31).

Zhang et al. also developed an ML algorithm-based model to predict bone metastasis in PCa patients. 211 patients diagnosed with PCa were randomized into a training group (n = 169; 80.1%) and a validation group (n = 42; 19.9%). 3D ROIs were cropped to extract radiomics features using PyRadiomics and DTL features using the ResNet 50. Pathognomonic features were extracted from H&E stained pathology images. To determine the optimal set of accuracy-based features, LASSO regression was employed. The most effective predictive model, which integrated radiomic features, DTL features, and pathognomonic features using a support vector machine, achieved an AUC of 0.93 (95% CI, 0.854 – 1.000). Further studies incorporating a larger sample size and whole slide images may potentially improve predictive accuracy (32). The aforementioned studies that have investigated CRPC-free survival using ML methods are summarized in **Table 3**.

**Table 3:** Metastasis-free survival using ML methods.

Site	Reference	Year	AI Model	Patients	Parameters	Predicted Outcomes
Lymph nodes	Hou et al. (26)	2021	RF	401 PCa patients (280 training/validation/71 test/50 external set)	Clinicopathological features, DTLR for extracting MRI radiomic features, radiologists’ interpretation	Pelvic LN metastasis model could have spared 59.6% of ePLNDs at the cost of missing only 1.7% of pelvic LN metastasis cases, outperforming the current MKSCC and Briganti nomograms.
	Wang et al. (27)	2023	RF, NBC, XGB, GBM, LR, DT	24,470 intermediate- and high-risk PCa patients	Clinicopathological features	Gradient boosting machine model demonstrated the highest prediction performance, with an F1 score of 0.838 and AUC of 0.804.
	Cysouw et al. (28)	2021	RF	76 intermediate- and high-risk PCa patients who underwent a preoperative [^18^F]DCFPyL PET-CT and RARP c ePLND	Radiomic features of PET-CT extracted with RaCaT software	The model predicted LNI (AUC 0.86 ± 0.15, *p* < 0.01), nodal or distant metastasis (AUC 0.86 ± 0.14, *p* < 0.01), Gleason score (0.81 ± 0.16, *p* < 0.01), and ECE (0.76 ± 0.12, *p* < 0.01). The AUCs were higher compared to standard PET metrics.
Bone	Liu et al. (31)	2021	DT, RF, MLP, LR, NBC, XGB	207,137 PCa patients of SEER database	Clinicopathological features	XGB model had the best predictive performance (AUC = 0.962, accuracy = 0.884, sensitivity = 0.906, and specificity = 0.879).
	Zhang et al. (32)	2024	LR, NBC, SVM, kNN, ExtraTrees, MLP, LightGBM, AdaBoost, XGBoost	211 PCa patients	Clinicopathological features, MRI radiomics features extracted with PyRadiomics and DTL features extracted with ResNet 50	The most effective predictive model, combining radiomic features, DTL features, and pathognomonic features using the SVM model, provided an AUC value of 0.93 (95% CI, 0.854 – 1.000).

RF, Random Forest Classifier; NBC, Naïve Bayes Classifier; XGB, Extreme Gradient Boosting; GBM, Gradient Boosting Machine; LR, Logistic Regression; DT, Decision Tree; MLP, Multilayer Perceptron; SVM, Support Vector Machine; kNN, k-Nearest Neighbor; AdaBoost, Adaptive Boosting.

## 6 Overall survival

### 6.1 Metastatic disease

Saito et al. used data from 340 metastatic PCa patients who received ADT as the initial treatment. 207 (60.9%) patients were used for the training set, and 103 (30.3%) patients were used for the test set. Random survival forest was used for ML survival analysis using the most important features, which included pretreatment lactic acid dehydrogenase (LDH) and alkaline phosphatase (ALP) levels four months following initial treatment. The model enabled patient grouping into three groups according to OS and cancer-specific survival prognoses. First, a very poor prognosis group with high pretreatment LDH (≥248.5 IU/L), in which approximately 70% of patients would expire within five years. The group with LDH <248.5 IU/L was further stratified into two groups based on post-treatment ALP level. The group with a high ALP level (≥326.5 IU/L) was an intermediate-risk group and had a 5-year survival rate of approximately 70%. The group with a low LDH level (<248.5 IU/L) prior to treatment and a low ALP level following treatment (<326.5 IU/L) had a very good prognosis, with a 5-year survival rate exceeding 90%. The C-index was 0.85 for both OS and CSS. The RSF model did not satisfactorily predict the prognosis of non-metastatic PCa patients, and further large-scale data and analysis focusing on clinical progression is warranted (33).

Anderson et al. examined 438 patients with mCRPC who experienced SREs and required radiotherapy or surgery. They developed six models using GBM techniques to estimate the probability of survival at 1, 2, 3, 4, 5, and 10 years after SRE treatment. The AUC values for these models ranged from 0.73 to 0.86, and the Brier scores were consistently below 0.20, indicating strong predictive accuracy. Positive survival indicators included younger age at metastasis diagnosis, PSA levels under ten ng/mL, a slow or stable rise in ALP levels, radiotherapy, and hormonal or chemotherapy treatments. Conversely, negative indicators were older age at diagnosis, PSA levels over 10 ng/mL, rapid increases in ALP levels, and being treatment-naïve. Decision curve analysis, which evaluates the net benefit of a clinical decision-making tool across various threshold probabilities, demonstrated that using these models can lead to optimal outcomes. Even though the study was limited by missing data, the model has the potential to be improved by the inclusion of additional demographic and laboratory values and external validation (34).

### 6.2 According to treatment modalities

Koo et al. analyzed data from 7,267 patients diagnosed with PCa and developed the SCaP (Severance Study Group of Prostate Cancer) Survival Calculator, which predicted 5- and 10-year survival rates of progression-free survival to CRPC, cancer-specific survival, and OS according to various initial treatment modalities including active surveillance, radical prostatectomy, radiation therapy with and without ADT, and ADT alone. When comparing the Cox regression model, multilayer perceptron, Multilayer perceptron (MLP) for N-year survival prediction, and long short-term memory (LSTM), the LSTM model showed the highest C-indices and AUC. Specifically, the C-index and AUC for LSTM prediction 10-year CRPC progression were 0.914 (0.890 – 0.928) and 0.920 (0.899–0.936), respectively. A web-based decision-making support system was developed, in which individual data such as age, height, weight, PSA, prostate volume, positive core numbers, maximal core percentage, Gleason score, Charlson Comorbidity index, performance status, TNM stage, secondary primary malignancy, and past medical history can be used to automatically compare survival outcomes according to each treatment modality. This simple yet accurate calculator can support the best treatment modality for individual patients. The limitation was the inclusion of patients from a single Asian ethnic background (35).

Lim et al. performed an external validation for this model by using data from 4,415 patients from three institutions. By using SCaP, the AUCs of 5-year CRPC-free survival, cancer-specific survival, and OS outcomes were AUCs of 0.962, 0.944, and 0.884, respectively. For 10-year outcomes, AUCs were 0.959, 0.928, and 0.854, respectively. The results outperformed the developmental model, validating the generalizability of the SCaP calculator. The limitation was that the data included patients who were treated over a long time span, during which treatment modalities and systemic agents had considerably improved (36).

### 6.3 Utilization of PET-CT

Polymeri et al. suggested an AI-based PET-CT coefficient could predict cancer-specific survival. They examined 285 patients who had performed ^18^F-choline PET-CT for newly diagnosed high-risk PCa, defined as PSA > 20ng/mL, and/or cT3, and/or Gleason score 8–10, with normal or inconclusive bone scans. The exclusion criteria were hormone therapy prior to PET-CT or PSA ≥ 150 ng/mL. Patients with no evidence of metastasis (n = 219, 76.8%) received curative treatment with either radical prostatectomy or radiation therapy. Patients who had metastasis (n = 66, 23.2%) received ADT based on PET-CT as a palliative setting. The AI-based model suggested by Mortensen et al. was used to automatically segment the prostate from PET and CT images (37). The AI-based algorithm automatically produced three volumetric measurements, including lesion volume, total lesion uptake, and the fraction of abnormal standardized uptake value (SUV) voxels to the total prostate volume, and was significantly associated with cancer-specific survival for patients receiving palliative treatment (*p* = 0.008, 0.02, and 0.005, respectively). The volume-based measurements performed better predictability compared to SUV_max_. This automated AI-based model provided reproducible information regarding the entire tumor, in contrast to the Gleason score, in which grading is highly subjective with interobserver variability. The limitations of this study include short-term follow-up, a small patient population, and the use of conventional PET-CT scans (38). The aforementioned studies that have investigated OS using ML methods are summarized in **Table 4**.

**Table 4:** Overall survival using ML methods.

Author/Reference	Year	AI Model	Patients	Parameters	Predicted Outcomes
Saito et al. (33)	2023	RSF	340 PCa patients who received ADT as initial treatment	Clinicopathological features, incorporating lab results	Patients with LDH < 248.5 IU/L and a low LDH level (< 248.5 IU/L) had a very good prognosis, with a 5-year survival rate exceeding 90%. The C-index was 0.85 for both OS and CSS.
Anderson et al. (34)	2022	GBM	438 mCRPC patients with SREs requiring treatment	Clinicopathological features	The models predicting 1-, 2-, 3-, 4-, 5-, and 10-year survival after treatment exhibited acceptable calibration, accuracy (Brier scores < 0.20), and classification ability (AUCs > 0.73).
Koo et al. (35)	2020	MLP, MLP-N, LSTM ANN models	7,267 PCa patients	Clinicopathological features, initial treatment modalities	LSTM model showed the highest C-indices and AUC. Specifically, the C-index and AUC for LSTM prediction 10-year CRPC progression were 0.914 (0.890 – 0.928) and 0.920 (0.899–0.936), respectively.
Lim et al. (36)	2021	LSTM ANN calculator by Koo et al. (35)	4,415 PCa patients	Clinicopathological features, initial treatment modalities	By using SCaP, the AUCs of 5-year CRPC-free survival, cancer-specific survival, and OS outcomes were AUCs of 0.962, 0.944, and 0.884, respectively.
Polymeri et al. (38)	2021	AI-based PET-CT automated analysis by Mortensen et al. (37)	145 PCa patients (training)/285 PCa patients (test)	Clinicopathological features, AI-based PET-CT automated analysis	Three volumetric measurements (lesion volume, total lesion uptake, and the fraction of abnormal standardized uptake value voxels to the total prostate volume) were associated with cancer-specific survival (*p* = 0.008, 0.02, and 0.005, respectively).

RSF, Random Survival Forest; GBM, Gradient Boosting Machine; MLP, Multilayer Perceptron; LSTM, Long Short-Term Memory; ANN, Artificial Neural Network.

## 7 Pitfalls of ML in the use of patient data

ML holds immense promise in revolutionizing PCa survival predictions and personalized treatment plans that go beyond traditional approaches. At the same time, data security, patient privacy, algorithmic bias, and the legal and ethical framework governing the use of data present significant obstacles that need to be addressed for successful integration into clinical practice (39). Empirical studies and domain-specific guidelines do not prescribe a strict rule for the optimal number of patients required to ensure generalizability of results. Instead, the optimal sample size depends on several factors, including model complexity, data variability, feature dimensionality, and the specific task at hand. More importantly, cross-validation techniques and external validation using independent datasets are critical for evaluating a model’s performance and generalizability, regardless of sample size. Caution is advised when interpreting studies that lack external validation, as they may not reliably reflect real-world scenarios. Additionally, potential biases and inequalities in algorithms developed using PCa populations characterized by heterogeneity and ethnic diversity must be carefully addressed. To fully leverage the potential of machine learning in healthcare, physicians and researchers must strike a balance between embracing technological advancements and exercising caution, fostering the development of an equitable and innovative healthcare system.

## 8 Summary and outlook

Survival prediction of PCa can be assessed from various aspects, each carrying significant clinical importance. The advent and improvement of AI are enabling the development of newer prediction models and nomograms that are non-inferior to or even superior to conventional traditional statistical-based methods. With advancements in ML techniques, alongside the growth of AI-focused companies and computational techniques, more precise and efficient ML models are being developed. This is reflected in the increasing number of articles published regarding the scope of this review – of the 20 key references shown in the tables, three (15.0%) were published before 2020 and 17 (85.0%) after 2020. These trends underscore the expanding role of ML in predicting PCa survival, thereby advancing personalized medicine.

Regarding PCa survival, AI can be applied across pathology, radiology, and electronic medical records. From a physician’s point of view, the prediction of LN metastasis can assist in the decision for lymphadenectomy and its extent. Metastasis-free survival prediction can be informative when deciding whether to provide adjuvant therapies. Specifically for bone metastasis-free survival, the identification of high-risk patients can allow for the implementation of preventive measures against the development of SREs. Regarding BCR prediction, data from prostate biopsy samples and prostate MRIs can be utilized to decide and communicate with patients to ensure the optimal treatment timepoint and modality.

To develop a more precise ML-based algorithm, several key strategies should be employed, including 1) enhancing the quality of data preparation, 2) selecting an optimal algorithm aligned with the study objective, 3) optimizing hyperparameters, 4) refining model architecture, 5) addressing overfitting and underfitting issues, and 6) improving computational power. Combining these essential strategies may systematically refine the model and improve its predictive power. When applying ML-based algorithms to real-world practice, it is crucial to address potential biases and inequalities, particularly those arising in algorithms trained on heterogeneous and ethnically diverse PCa populations. External validation across diverse countries, ethnicities, and medical centers can bolster the reliability and generalizability of these models, facilitating their inclusion in clinical guidelines and routine practice. Achieving this requires collaboration with industries such as health insurance providers and innovative startups, which can support the widespread adoption of these advanced tools.

Future studies could explore the prediction of treatment discontinuation and complications, which would aid in choosing treatment modalities for metastatic PCa patients. In addition, future AI models could consider the incorporation of newer imaging modalities, such as PSMA PET-CT, in the prediction of survival, which could potentially change the armamentarium of PCa treatment. Lastly, studies that predict genetic mutation status using AI and ML could be anticipated.
